# Reverse vaccinology and immunoinformatics identify multi-epitope vaccine candidates against molluscum contagiosum virus

**DOI:** 10.1093/biomethods/bpag045

**Published:** 2026-08-13

**Authors:** Shahrazad A Khalaf, Musaab M Jasim, Younus Jasim, Murtada Naser

**Affiliations:** Department of Forensic Sciences, College of Science, University of Diyala, Baqubah 32001, Iraq; Department of Forensic Sciences, College of Science, University of Diyala, Baqubah 32001, Iraq; Department of Medical Laboratory Technologies and Artificial Intelligence, High Institute of Health Technologies and Artificial Intelligence, Northern Technical University, Mosul 41000, Iraq; Division of BioInvasions, Global Change and Macroecology, Department of Botany and Biodiversity Research, University of Vienna, Vienna 1030, Austria

**Keywords:** molluscum contagiosum virus, reverse vaccinology, immunoinformatics, multi-epitope vaccine, TLR4

## Abstract

Molluscum contagiosum virus (MCV) is a human-specific poxvirus responsible for a common contagious skin infection, yet no licensed prophylactic vaccine is available. Therefore, this study aimed to identify potential vaccine candidates against MCV using integrated reverse vaccinology and immunoinformatics approach. Candidate proteins involved in immune evasion, viral entry, and virion assembly were assessed for physicochemical properties, transmembrane topology, antigenicity, allergenicity, and toxicity. Linear B-cell, cytotoxic T-lymphocyte (CTL), and helper T-lymphocyte (HTL) epitopes were predicted and prioritized based on antigenicity, safety, HLA-binding affinity, and worldwide population coverage. The selected epitopes were assembled into a multi-epitope vaccine construct, followed by physicochemical characterization, secondary and tertiary structure prediction, and molecular docking analysis with Toll-like receptor 4 (TLR4). Eight candidate proteins were selected for immunoinformatics analysis, of which six were predicted to be antigenic. Five HTL epitopes, five CTL epitopes, and three non-toxic antigenic B-cell epitopes were selected, providing an estimated worldwide population coverage of 80.69%. The final 351-amino acid construct had a predicted molecular weight of 37.48 kDa, an instability index of 33.29, and a hydrophilic GRAVY value of −0.201. AlphaFold 3 produced a structurally organized model, while docking predicted a favorable interaction with TLR4 (score, −190.46) supported by hydrogen bonds, salt bridges, and electrostatic interactions. The vaccine construct exhibited favorable physicochemical properties, broad population coverage, structural stability, and strong interaction with TLR4, suggesting its potential as a prophylactic vaccine candidate. Nevertheless, further *in vitro* and *in vivo* experimental validation is required to confirm its safety and protective efficacy.

## Introduction

Molluscum contagiosum is a common viral infection of the skin caused by the Molluscum contagiosum virus (MCV), a member of the Poxviridae family. It appears as small, dome-shaped papules of the skin color with central umbilication. Children, sexually active adults, and immunocompromised individuals are primarily affected [1, 2]. Although generally considered a self-limiting disease, persistent lesions can cause cosmetic concern, discomfort, autoinoculation, and significant psychosocial burden, especially among immunocompromised patients and those with extensive disease [3–5]. MCV is one of the most prevalent viral skin pathogens worldwide and is transmitted primarily through direct skin-to-skin contact, autoinoculation, and contaminated fomites. Increased disease severity and prolonged persistence have been reported in patients with HIV infection, atopic dermatitis, and other conditions associated with impaired immune function [4, 6].

The MCV genome is approximately 190 kb of linear double-stranded DNA. It contains more than 160 proteins involved in viral replication, virion assembly, host interaction, and immune modulation [7, 8]. MCV establishes a localized epidermal infection with little inflammation, distinguishing it from many other poxviruses, which allows it to persist within the host and evade host clearance [8]. The ability of MC159 and MC160 to promote survival signaling, suppress apoptosis, and inhibit the innate immune response likely plays a role in viral persistence within the host. Indeed, MCV encodes numerous immune-modulatory proteins that collectively enable the virus to evade host immune surveillance and establish persistent infection. For example, viral proteins such as MC159 and MC160 disrupt apoptotic pathways, NF-κB signaling, interferon responses, and cGAS-STING-mediated innate immune activation, thus creating a favorable environment for viral replication and dissemination within the host by suppressing antiviral immune responses in epidermal keratinocytes [7, 8].

Despite the clinical burden associated with MC, there is no licensed vaccine for the prevention of MCV infection. Current therapies, including curettage, cryotherapy, cantharidin, potassium hydroxide, and topical nitric oxide-releasing agents, only remove lesions and do not provide long-term protective immunity [5, 9]. Recent therapeutic developments such as berdazimer sodium gel have shown promising clinical efficacy; however, these remain therapeutic rather than prophylactic and do not provide durable immune protection against reinfection [10, 11]. Recent advances in computational biology have accelerated vaccine discovery through reverse vaccinology and immunoinformatics approaches. Unlike conventional vaccine development strategies, reverse vaccinology utilizes genomic and proteomic information to identify conserved, immunogenic, and non-allergenic vaccine targets without requiring pathogen cultivation. This strategy has substantially reduced the time and cost associated with vaccine development and has been successfully applied to several bacterial and viral pathogens [9, 12, 13].

Multi-epitope vaccine design integrates B-cell, cytotoxic T lymphocyte (CTL), and helper T lymphocyte (HTL) epitopes into a single construct capable of stimulating both humoral and cellular immune responses. Combined with structural bioinformatics, molecular docking, and molecular dynamics simulation, immunoinformatics-based vaccine development provides a rational framework for developing next-generation vaccines [13, 14].

Therefore, this study employed a comprehensive reverse vaccinology and immunoinformatics pipeline to identify promising antigenic proteins from MCV and construct a multi-epitope vaccine candidate with favorable immunogenic, physicochemical, and structural characteristics. The designed vaccine was further evaluated through structural validation, molecular docking with Toll-like receptor 4 (TLR4), and normal mode analysis to assess its potential as a prophylactic vaccine candidate against MCV infection.

## Materials and methods

### Study design

**Figure 1:**
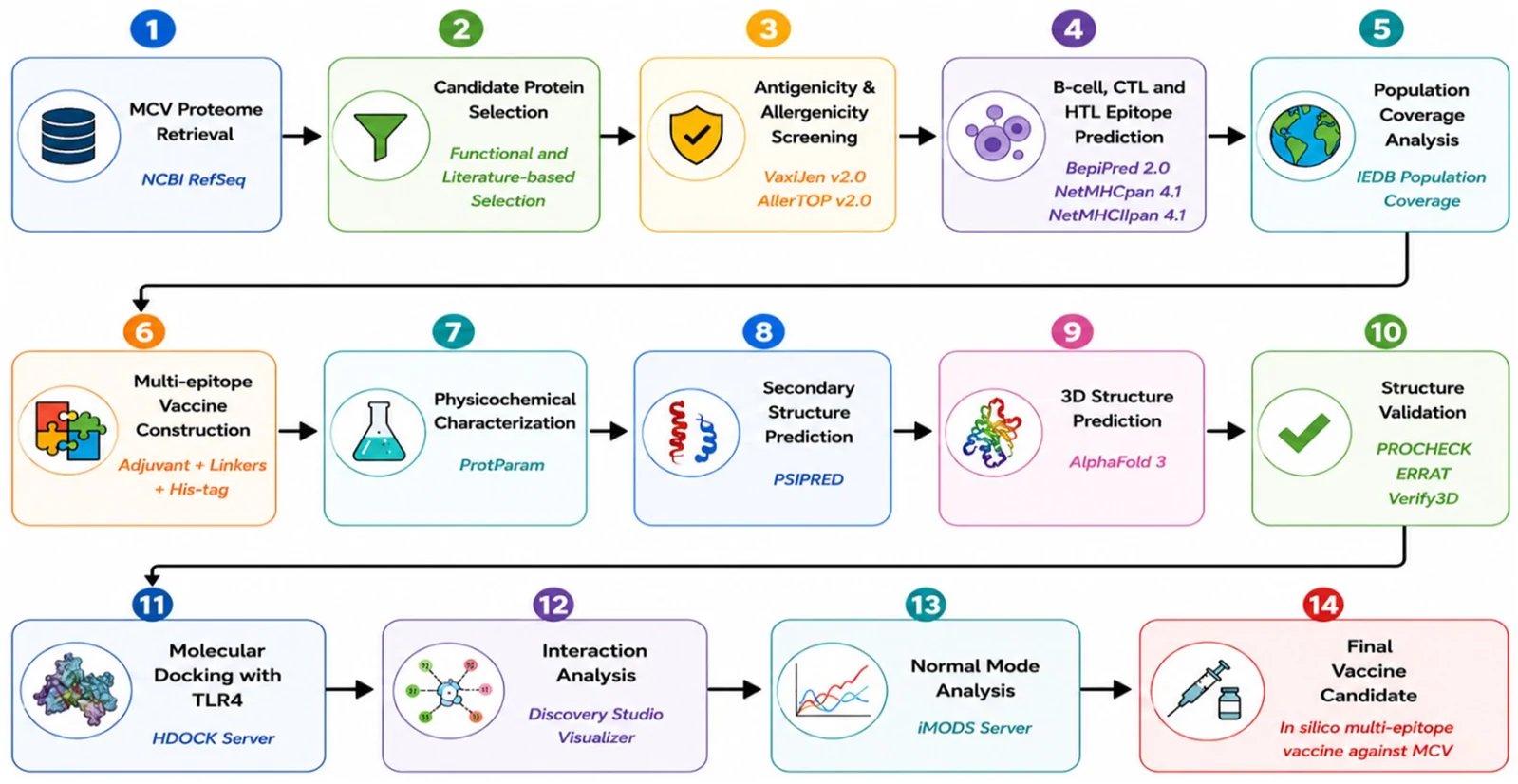
Schematic workflow of the reverse vaccinology strategy used for multi-epitope vaccine design against MCV

This study employed an integrated reverse vaccinology and immunoinformatics strategy to identify potential vaccine candidates against MCV by systematically screening the viral proteome using computational approaches [15]. The workflow consisted of proteome retrieval, candidate protein prioritization, physicochemical characterization, transmembrane topology prediction, antigenicity assessment, allergenicity evaluation, toxicity prediction, B-cell and T-cell epitope identification, population coverage analysis, multi-epitope vaccine construction, structural modeling, molecular docking, and interaction analysis [16]. The proposed computational pipeline was designed to identify predicted highly antigenic, non-allergenic, non-toxic, and structurally stable vaccine candidates suitable for future experimental validation [16]. The computational resources in this study were chosen for four reasons: they are peer-reviewed, widely benchmarked, publicly accessible, and capable of producing standardized outputs for sequential vaccine-design analyses. Tools received preference when they included virus-specific models, training datasets informed by experiments, broad coverage of HLA alleles, or confidence metrics suited to the analytical step at hand. The schematic workflow of the reverse vaccinology strategy used for multi-epitope vaccine design against MCV is shown in Fig. 1.

### Retrieval of the viral proteome

The complete reference proteome of MCV subtype 1 (MCV-1) was retrieved from the National Center for Biotechnology Information (NCBI) RefSeq database in FASTA format [17]. Protein accession numbers, amino acid sequences, gene annotations, and functional descriptions were collected for all annotated viral proteins available in the RefSeq repository [17]. The retrieved dataset comprised 163 annotated proteins and served as the initial dataset for subsequent reverse vaccinology analysis. The MCV-1 RefSeq proteome was chosen as a curated, complete reference dataset for reproducible proteome-wide screening. Still, it may not fully represent MCV-2, MCV-3, or other genetic variants. The predictions therefore apply specifically to MCV-1; before inferring broader cross-subtype protection, the selected proteins and epitopes should be assessed for conservation across additional MCV genotypes. RefSeq was chosen for its curated, non-redundant reference sequences and standardized functional annotations.

### Candidate protein selection

Candidate proteins were selected according to their reported biological roles in viral pathogenesis, with particular emphasis on proteins involved in immune evasion, viral entry, membrane fusion, and virion assembly, as these proteins are more likely to induce protective immune responses [8]. Functional annotation available in the NCBI RefSeq database and published literature was used to prioritize proteins with potential immunological relevance for downstream analysis [8, 17]. Proteins with predicted roles in essential viral functions and host–pathogen interactions were prioritized because they are more likely to be accessible to immune recognition and represent suitable targets for vaccine development. Eight candidate proteins were selected for detailed immunoinformatics analysis based on these selection criteria.

### Physicochemical characterization

The physicochemical properties of the selected proteins were analyzed using the ProtParam program available through the ExPASy server [14]. Protein length, molecular weight, theoretical isoelectric point (pI), instability index, aromatic amino acid content, aliphatic index, and grand average of hydropathicity (GRAVY) were calculated to evaluate protein stability and biochemical characteristics [14]. Proteins predicted to possess favorable physicochemical properties were retained for subsequent analysis.

### Transmembrane topology prediction

The membrane topology of each candidate protein was predicted using the DeepTMHMM server, which applies protein language models and deep neural networks to identify transmembrane helices, signal peptides, and protein topology with high accuracy [18]. Proteins containing extracellular or membrane-associated regions were considered high-priority vaccine candidates because surface-exposed proteins are more accessible to host immune recognition by the host immune system [18].The predicted topology of each protein was subsequently incorporated into the candidate prioritization process. DeepTMHMM was used to predict transmembrane helices, signal peptides, and protein topology through a single language-model-based framework.

### Antigenicity prediction

The antigenicity of the selected candidate proteins was evaluated using the VaxiJen v2.0 server, an alignment-independent prediction tool based on the auto-cross covariance (ACC) transformation of protein sequences [19]. A threshold value of 0.40 was applied to the ACC-derived VaxiJen antigenicity score for viral proteins, as recommended by the tool developers. Proteins with score above 0.40 were classified as probable antigens and retained for subsequent immunoinformatics analysis, whereas proteins scoring below this threshold were excluded [19]. VaxiJen was chosen because its viral antigenicity model does not depend on sequence alignment, making it suitable for proteins without experimentally characterized homologues.

### Allergenicity prediction

The allergenic potential of the antigenic proteins was evaluated using the AllerTOP v2.0 server, which predicts allergenicity based on amino acid physicochemical properties and machine-learning algorithms [20]. Only proteins predicted as probable non-allergens were retained for downstream epitope prediction to minimize the risk of vaccine-induced allergic reactions [20].

### Prediction of linear B-cell epitopes

Linear B-cell epitopes were predicted using the BepiPred-2.0 server available through the Immune Epitope Database (IEDB), which combines hidden Markov models with propensity scale methods to identify antibody-accessible regions within protein sequences [21]. Predicted epitopes were selected based on their prediction scores and sequence continuity before being subjected to further immunological evaluation [21]. The antigenicity of each predicted B-cell epitope was subsequently evaluated using VaxiJen v2.0, and only antigenic epitopes were retained for downstream analysis [19]. BepiPred 2.0 was chosen for its integration with IEDB, as well as its use of experimentally characterized epitope data during development.

### Toxicity prediction

The toxicity of the selected antigenic B-cell epitopes was assessed using ToxinPred2, which predicts peptide toxicity using machine-learning algorithms trained on experimentally validated toxic and non-toxic peptides [22]. Only epitopes predicted to be non-toxic were considered suitable for inclusion in the final vaccine construct.

### Prediction of cytotoxic T-lymphocyte epitopes

Cytotoxic T-lymphocyte (CTL) epitopes were predicted using the NetMHCpan 4.1 EL server, which predicts peptide binding affinity to human MHC class I molecules based on artificial neural network algorithms trained with both binding affinity and naturally eluted ligand datasets [23]. Nine-mer peptide sequences were screened against representative human HLA class I alleles, and peptides exhibiting the lowest percentile ranks were considered strong binders [23]. Predicted CTL epitopes with high binding affinity and broad HLA allele representation were selected for subsequent vaccine construction.

### Prediction of helper T-lymphocyte epitopes

Helper T-lymphocyte (HTL) epitopes were predicted using the IEDB NetMHCIIpan 4.1 EL prediction server, which estimates peptide binding affinity to human MHC class II molecules using artificial neural networks trained on experimentally validated peptide–HLA binding datasets [23]. Peptides of appropriate length were screened against representative HLA-DR alleles, and candidate epitopes exhibiting the lowest percentile rank values were considered strong binders and prioritized for further evaluation [23]. Only high-affinity HTL epitopes with broad HLA allele coverage were retained for subsequent vaccine construction.

### Population coverage analysis

The worldwide population coverage of the selected CTL and HTL epitopes was evaluated using the IEDB Population Coverage tool to estimate the proportion of individuals predicted to respond to the selected HLA–epitope combinations [24]. The analysis was performed using the combined HLA class I and class II alleles associated with the selected epitopes to estimate global population coverage, the average number of epitope hits per individual, and PC90 values [24]. Population coverage analysis was performed to ensure that the proposed vaccine construct would provide broad immunological applicability across diverse human populations. NetMHCpan platforms were included because their pan-allelic models can predict binding across a broad range of HLA molecules and draw on naturally eluted ligand data.

### Construction of the multi-epitope vaccine

The final vaccine construct was designed by assembling the selected HTL, CTL, and B-cell epitopes according to their predicted immunological properties. The 50S ribosomal protein L7/L12 was incorporated at the N-terminus as an immunostimulatory adjuvant because of its reported ability to enhance innate immune responses through Toll-like receptor stimulation [25]. The adjuvant was connected to the first epitope using the rigid EAAAK linker to minimize structural interference between functional domains [26]. Adjacent HTL epitopes were linked using the GPGPG linker to facilitate efficient antigen presentation by MHC class II molecules [26]. CTL epitopes were connected using the AAY linker to promote efficient proteasomal processing and MHC class I presentation [26]. Linear B-cell epitopes were joined using the KK linker to preserve epitope flexibility and improve antibody accessibility [26]. Finally, a 6 × His purification tag was incorporated at the C-terminus of the construct to facilitate recombinant protein purification and detection during future experimental studies.

### Physicochemical evaluation of the vaccine construct

The physicochemical characteristics of the final vaccine construct were analyzed using the ExPASy ProtParam server [14]. Protein length, molecular weight, theoretical isoelectric point (pI), amino acid composition, instability index, aliphatic index, extinction coefficient, estimated half-life, and grand average of hydropathicity (GRAVY) were calculated to evaluate the biochemical stability and suitability of the designed vaccine construct [14]. The obtained parameters were used to assess the overall stability and physicochemical behavior of the recombinant vaccine.

### Secondary structure prediction

The secondary structural elements of the designed vaccine construct were predicted using the PSIPRED server, which predicts α-helices, β-strands, and random coils based on neural-network analysis of position-specific scoring matrices generated by PSI-BLAST [27]. Secondary structure prediction was performed to evaluate the structural organization of the vaccine construct prior to three-dimensional (3D) structural structure prediction [27].

### 3D structure prediction

The 3D structure of the designed multi-epitope vaccine construct was predicted using AlphaFold 3, an artificial intelligence-based protein structure prediction system that integrates deep learning algorithms to generate highly accurate structural models from amino acid sequences [28]. The predicted structural model was used to evaluate the overall folding pattern and spatial organization of the vaccine construct prior to molecular docking analysis [28]. In addition, the Predicted Aligned Error (PAE) matrix generated by AlphaFold 3 was analyzed to assess the confidence of residue–residue positioning within the predicted protein structure [28]. AlphaFold 3 was chosen for its predicted structural model and its residue-pair confidence estimates, provided through the PAE output.

### Molecular docking

Molecular docking was performed using the HDOCK server, a hybrid protein-protein docking platform that integrates template-based modeling with free docking algorithms for the prediction of receptor-ligand complexes. The crystal structure of the human TLR4 (PDB ID: 4G8A, Chain A) served as the receptor, and the vaccine construct was used as the ligand for docking analysis. Multiple models were automatically generated by the HDOCK server, and the highest-ranked model, corresponding to the lowest docking score for further interaction analysis. Docking performance was evaluated based on the docking score, confidence score, template coverage, sequence identity, and predicted binding interactions. HDOCK was chosen because it combines template-based protein–protein docking with a template-free approach.

### Protein–protein interaction analysis

The intermolecular interactions within the selected vaccine-TLR4 docking complex were analyzed using BIOVIA Discovery Studio Visualizer, which enables visualization and analysis of protein–protein interfaces and non-covalent molecular interactions (Dassault Systèmes BIOVIA, 2021). Hydrogen bonds, salt bridges, electrostatic interactions, and amino acid residues at the binding interface were identified and characterized to assess the stability of the predicted vaccine-receptor complex. This interaction profile was used to support the predicted immunological recognition of the designed vaccine through TLR4-mediated innate immune activation.

## Results

### Retrieval of the MCV proteome

The complete MCV-1 reference proteome was obtained from the NCBI RefSeq database and contained 163 annotated structural and non-structural proteins. This set served as the starting point for computational screening and prioritization of vaccine targets. Table S1 lists all proteins along with their RefSeq accession numbers, gene designations, sequence lengths, molecular weights, and available functional annotations.

The complete proteome comprising 163 proteins is provided in Table S1.

### Candidate protein selection

Application of the predefined selection criteria resulted in the identification of eight candidate proteins with potential immunological relevance. These proteins (MC005L, MC008L, MC016L, MC057L, MC114R, MC159L, MC160L, and MC163R) were retained for subsequent immunoinformatics analysis. These proteins were considered promising candidates because of their potential involvement in host–virus interactions and their likelihood of eliciting protective immune responses. The selected proteins and the rationale for their inclusion are presented in Table 1.

**Table 1:** Initial candidate proteins selected for reverse vaccinology analysis based on their reported biological functions.

No.	RefSeq accession	Gene	Biological role	Selection rationale^a^	References
**1**	NP_043956.1	MC005L	Immune evasion	Selected because it is predicted to interfere with the NF-κB/IKK signaling pathway, contributing to viral immune evasion.	[29]
**2**	NP_043959.1	MC008L	Immune evasion	Selected because it suppresses host innate immune responses through inhibition of NF-κB activation.	[30]
**3**	NP_043967.1	MC016L	Viral entry/fusion	Selected as a conserved component of the poxvirus entry–fusion complex required for efficient host cell entry.	[31]
**4**	NP_044008.1	MC057L	Viral entry/fusion	Selected because it participates in the viral entry–fusion machinery essential for infection.	[32]
**5**	NP_044065.1	MC114R	Structural protein	Selected because it is predicted to participate in early virion morphogenesis and viral particle assembly, particularly during viral crescent formation, a critical step in poxvirus maturation.	[33]
**6**	NP_044110.1	MC159L	Immune evasion	Selected because it encodes a viral FLIP protein that inhibits apoptosis and modulates inflammatory signaling pathways.	[16,34]
**7**	NP_044111.1	MC160L	Immune evasion	Selected because it functions as an immune modulatory protein involved in suppression of host antiviral responses.	[34,35]
**8**	NP_044114.1	MC163R	Immune evasion	Selected because it acts as an anti-apoptotic protein that promotes viral persistence within infected cells.	[36]

a Candidate proteins were selected according to their reported involvement in viral entry, immune evasion, or virion assembly prior to downstream reverse vaccinology analyses.

### Physicochemical characterization of candidate proteins

Physicochemical characterization was performed before epitope prediction to evaluate the biochemical properties and predicted structural stability of the selected candidate proteins. The proteins varied in molecular weight, theoretical isoelectric point (pI), instability index, and grand average of hydropathicity (GRAVY). The theoretical pI is the pH at which a protein carries no net electrical charge. GRAVY, by contrast, describes the protein’s overall hydropathic character: positive values indicate greater hydrophobicity, whereas negative values indicate greater hydrophilicity. According to their instability index values, MC005L, MC016L, and MC163R were classified as stable; the other proteins were predicted to be unstable under *in vitro* conditions. Table 2 summarizes the detailed physicochemical properties.

**Table 2:** Physicochemical properties of the selected MCV candidate proteins predicted using the ProtParam tool.

Gene	Length (aa)	Molecular weight (kDa)	Theoretical isoelectric point (pI)	Aromaticity	Instability index	Stability	GRAVY (hydropathicity index)
**MC005L**	85	8.90	4.83	0.024	38.47	Stable	0.348
**MC008L**	176	19.23	8.06	0.068	47.84	Unstable	0.240
**MC016L**	213	22.89	9.85	0.047	37.59	Stable	0.309
**MC057L**	108	12.45	9.95	0.074	44.86	Unstable	−0.110
**MC114R**	304	33.24	4.90	0.049	51.37	Unstable	−0.070
**MC159L**	241	26.94	5.13	0.050	71.73	Unstable	−0.151
**MC160L**	371	38.63	4.47	0.040	47.21	Unstable	0.200
**MC163R**	620	65.90	7.87	0.048	35.89	Stable	−0.422

### Transmembrane topology prediction

The proteins were subjected to transmembrane topology analysis to check for the presence of membrane-spanning regions. DeepTMHMM prediction showed that three proteins (MC016L, MC057L, and MC163R) contained a single transmembrane helix, indicating membrane localization with predicted extracellular regions. These proteins were prioritized for subsequent immunoinformatics analysis. The proteins MC005L, MC008L, MC159L, and MC160L were predicted to be globular intracellular proteins without a transmembrane helix, while MC114R was kept for further analysis due to its known structural function without having a transmembrane domain. The full results of the topology predictions are in Table 3.

**Table 3:** Predicted transmembrane topology of the selected MCV candidate proteins using DeepTMHMM.

No.	Gene	RefSeq accession	Protein type	No. TM helices	Predicted topology	Signal peptide	Decision
**1**	MC005L	NP_043956.1	Globular	0	Intracellular (Inside)	No	Keep
**2**	MC008L	NP_043959.1	Globular	0	Intracellular (Inside)	No	Keep
**3**	MC016L	NP_043967.1	Transmembrane	1	Outside–TM–Inside	No	**High priority**
**4**	MC057L	NP_044008.1	Transmembrane	1	Inside–TM–Outside	No	**High priority**
**5**	MC114R	NP_044065.1	Globular	0	Intracellular (Inside)	No	Evaluate
**6**	MC159L	NP_044110.1	Globular	0	Intracellular (Inside)	No	Keep
**7**	MC160L	NP_044111.1	Globular	0	Intracellular (Inside)	No	Keep
**8**	MC163R	NP_044114.1	Transmembrane	1	Inside–TM–Outside	No	**High priority**

Note: Bold text indicates high-priority candidate proteins based on the predicted transmembrane topology.

### Antigenicity prediction

The antigenic potential of the selected candidate proteins was evaluated using the VaxiJen v2.0 server at a threshold value of 0.40. Six proteins exceeded the predefined threshold and hence were classified as probable antigens suitable for downstream vaccine design. Among these, MC057L showed the highest antigenicity score0.6098, followed by MC016L and MC005L 0.5625 and 0.5201, respectively, indicating their predicted antigenic potential. MC159L and MC163R were predicted as non-antigenic with scores of 0.3495 and 0.3632, respectively, and hence were not considered for epitope prediction analysis. MC114R had a score of 0.4036 which is on the border but was taken because it remains above the selected threshold. Full details of the scores and decisions are shown in Table 4.

**Table 4:** Antigenicity prediction of the selected MCV candidate proteins using VaxiJen v2.0 (threshold = 0.40).

No.	Gene	VaxiJen score	Prediction	Decision
**1**	MC005L	0.5201	Probable antigen	Keep
**2**	MC008L	0.4376	Probable antigen	Keep
**3**	MC016L	0.5625	Probable antigen	Keep
**4**	MC057L	0.6098	Probable antigen	Keep
**5**	MC114R	0.4036	Probable antigen	Keep (borderline)
**6**	MC159L	0.3495	Probable non-antigen	Exclude
**7**	MC160L	0.4854	Probable antigen	Keep
**8**	MC163R	0.3632	Probable non-antigen	Exclude

### Allergenicity prediction

The allergenic potential of the selected candidate proteins was evaluated using the AllerTOP v2.0 server. All eight selected proteins were predicted as probable non-allergens, suggesting a low predicted risk of inducing allergic responses. This result supports the suitability of the selected proteins for epitope prediction and subsequent vaccine construction. Although MC159L and MC163R were later excluded based on antigenicity assessment, the fact that they were also predicted as non-allergenic further confirmed the reliability of the computational screening process. Results of the allergenicity prediction are shown in Table 5.

**Table 5:** Allergenicity prediction of selected MCV candidate proteins using AllerTOP v2.0.

No.	Gene	RefSeq accession	AllerTOP prediction	Decision
**1**	MC005L	NP_043956.1	Probable non-allergen	Keep
**2**	MC008L	NP_043959.1	Probable non-allergen	Keep
**3**	MC016L	NP_043967.1	Probable non-allergen	Keep
**4**	MC057L	NP_044008.1	Probable non-allergen	Keep
**5**	MC114R	NP_044065.1	Probable non-allergen	Keep
**6**	MC159L	NP_044110.1	Probable non-allergen	Keep^a^
**7**	MC160L	NP_044111.1	Probable non-allergen	Keep
**8**	MC163R	NP_044114.1	Probable non-allergen	Keep^a^

a MC159L and MC163R passed the allergenicity screen, as both were predicted to be non-allergenic. They were later excluded from epitope prediction because their VaxiJen antigenicity scores fell below the predefined threshold of 0.40.

### Prediction of linear B-cell epitopes

The top MCV proteins had several antibody-accessible peptide regions with multiple linear B-cell epitopes. Nineteen linear B-cell epitopes were selected after filtering for prediction score and continuity of epitopes. The selected epitopes, from five proteins, ranged in length from 8 to 28 amino acids (MC005L, MC008L, MC016L, MC057L, MC114R, and MC160L). These epitopes were considered putative antibody-binding sites and served as the initial set for subsequent antigenicity and toxicity screening. Table 6 lists the filtered B-cell epitopes.

**Table 6:** Filtered linear B-cell epitopes selected for downstream immunoinformatics analyses.

Protein	Epitope ID	Start	End	Epitope sequence	Length (aa)	Average score
**MC005L**	E1	30	42	QMRLLSEEEKTSC	13	0.486
**MC005L**	E2	57	82	CRGGRQALLQARGEPASLGEVAGKGP	26	0.486
**MC008L**	E1	6	14	IVRDLGLAG	9	0.471
**MC008L**	E2	21	33	LSAAGLPQPERAC	13	0.471
**MC008L**	E3	47	56	FQEDYHVGET	10	0.471
**MC008L**	E4	72	82	STAGADAERAM	11	0.471
**MC008L**	E5	132	139	ICLCAREE	8	0.471
**MC016L**	E1	19	30	RKLSLYSTTNSV	12	0.475
**MC016L**	E2	89	107	GVDLEAASHEESRLERKCR	19	0.475
**MC016L**	E3	156	176	SLSQRPAFVPVDARRALCPGA	21	0.475
**MC016L**	E4	201	209	LRLRYQYAT	9	0.475
**MC057L**	E1	33	52	DAQARAAAVQARADEAPRARR	20	0.482
**MC114R**	E1	7	34	TDIANEYAVTTFSEDGYPCNKNYEITSG	28	0.513
**MC114R**	E3	148	163	ALRRRRAEQGSDNLAN	16	0.513
**MC114R**	E4	168	179	LFQKNPARAGTV	12	0.513
**MC114R**	E6	250	262	DDIKGLSSEVTSE	13	0.513
**MC160L**	E1	76	93	GMTRECAGRLLGHGFLSQ	18	0.507
**MC160L**	E3	231	253	CTSAGKGEDLATSDLTDSEPEDS	23	0.507
**MC160L**	E5	321	345	DANSSLMRTTSACTDSEPEDSAGPS	25	0.507

### Antigenicity assessment of B-cell epitopes

After the linear B-cell epitopes were identified, their antigenic potential was further evaluated. The VaxiJen v2.0 server was used to predict the antigenicity of the predicted B-cell epitopes; most of the predicted epitopes had antigenicity scores higher than the threshold, 0.40, and were therefore probable antigens. Among the selected peptides, several epitopes had high antigenicity scores. The highest was recorded for MC008L epitope E5 (ICLCAREE), at 1.4819. MC016L epitope E4 (LRLRYQYAT) followed with a score of 1.4631, while MC016L epitope E3 (SLSQRPAFVPVDARRALCPGA) scored 1.3313. By contrast, MC114R epitope E6 (DDIKGLSSEVTSE) and MC160L epitope E5 (DANSSLMRTTSACTDSEPEDSAGPS) were predicted to be non-antigenic, so they were excluded from further analysis. The antigenicity screening reduced the number of candidate epitopes and showed highly immunogenic peptides suitable for toxicity assessment and vaccine construction. The detailed antigenicity scores and selection decisions are provided in Table 7.

**Table 7:** Antigenicity assessment of filtered B-cell epitopes using VaxiJen v2.0.

Protein	Epitope ID	Epitope sequence	Length (aa)	VaxiJen score	Prediction	Decision
**MC005L**	E1	QMRLLSEEEKTSC	13	0.8020	Probable antigen	Selected
**MC005L**	E2	CRGGRQALLQARGEPASLGEVAGKGP	26	0.7855	Probable antigen	Selected
**MC008L**	E1	IVRDLGLAG	9	0.5494	Probable antigen	Selected
**MC008L**	E2	LSAAGLPQPERAC	13	0.5757	Probable antigen	Selected
**MC008L**	E3	FQEDYHVGET	10	0.7403	Probable antigen	Selected
**MC008L**	E4	STAGADAERAM	11	0.6866	Probable antigen	Selected
**MC008L**	E5	ICLCAREE	8	1.4819	Probable antigen	Selected
**MC016L**	E1	RKLSLYSTTNSV	12	0.6880	Probable antigen	Selected
**MC016L**	E2	GVDLEAASHEESRLERKCR	19	1.0952	Probable antigen	Selected
**MC016L**	E3	SLSQRPAFVPVDARRALCPGA	21	1.3313	Probable antigen	Selected
**MC016L**	E4	LRLRYQYAT	9	1.4631	Probable antigen	Selected
**MC057L**	E1	DAQARAAAVQARADEAPRARR	20	0.6421	Probable antigen	Selected
**MC114R**	E1	TDIANEYAVTTFSEDGYPCNKNYEITSG	28	0.7335	Probable antigen	Selected
**MC114R**	E3	ALRRRRAEQGSDNLAN	16	0.6641	Probable antigen	Selected
**MC114R**	E4	LFQKNPARAGTV	12	0.4388	Probable antigen	Selected
**MC114R**	E6	DDIKGLSSEVTSE	13	0.3353	Probable Non-antigen	Rejected
**MC160L**	E1	GMTRECAGRLLGHGFLSQ	18	−0.6700	Probable non-antigen	Rejected
**MC160L**	E3	CTSAGKGEDLATSDLTDSEPEDS	23	1.0290	Probable antigen	Selected
**MC160L**	E5	DANSSLMRTTSACTDSEPEDSAGPS	25	0.3617	Probable non-antigen	Rejected

### Toxicity prediction of selected B-cell epitopes

To ensure the safety of the proposed vaccine construct, all antigenic B-cell epitopes were evaluated using the ToxinPred2 server. Toxicity analysis demonstrated that only three epitopes were predicted to be non-toxic and therefore considered suitable for inclusion in the final vaccine design. These included the MC008L epitope E3 (FQEDYHVGET), the MC016L epitope E1 (RKLSLYSTTNSV), and the MC114R epitope E1 (TDIANEYAVTTFSEDGYPCNKNYEITSG). The remaining antigenic epitopes were predicted to possess toxic characteristics and were consequently excluded despite their favorable antigenicity scores. These findings highlight the importance of integrating both antigenicity and toxicity screening during epitope prioritization to ensure the selection of immunogenic peptides with an acceptable predicted safety profile. The complete toxicity prediction results are summarized in Table 8.

**Table 8:** Toxicity prediction of antigenic B-cell epitopes using ToxinPred2 (ML model).

Protein	Epitope ID	Epitope sequence	Length (aa)	VaxiJen score	ML score	ToxinPred2 prediction	Decision
**MC005L**	E1	QMRLLSEEEKTSC	13	0.8020	0.7600	Toxin	Rejected
**MC005L**	E2	CRGGRQALLQARGEPASLGEVAGKGP	26	0.7855	0.7000	Toxin	Rejected
**MC008L**	E1	IVRDLGLAG	9	0.5494	0.8000	Toxin	Rejected
**MC008L**	E2	LSAAGLPQPERAC	13	0.5757	0.7600	Toxin	Rejected
**MC008L**	E3	FQEDYHVGET	10	0.7403	0.5300	Non-Toxin	Selected
**MC008L**	E4	STAGADAERAM	11	0.6866	0.6300	Toxin	Rejected
**MC008L**	E5	ICLCAREE	8	1.4819	0.8300	Toxin	Rejected
**MC016L**	E1	RKLSLYSTTNSV	12	0.6880	0.5700	Non-toxin	Selected
**MC016L**	E2	GVDLEAASHEESRLERKCR	19	1.0952	0.8200	Toxin	Rejected
**MC016L**	E3	SLSQRPAFVPVDARRALCPGA	21	1.3313	0.7000	Toxin	Rejected
**MC016L**	E4	LRLRYQYAT	9	1.4631	0.6300	Toxin	Rejected
**MC057L**	E1	DAQARAAAVQARADEAPRARR	20	0.6421	0.6700	Toxin	Rejected
**MC114R**	E1	TDIANEYAVTTFSEDGYPCNKNYEITSG	28	0.7335	0.5800	Non-toxin	Selected
**MC114R**	E3	ALRRRRAEQGSDNLAN	16	0.6641	0.7200	Toxin	Rejected
**MC114R**	E4	LFQKNPARAGTV	12	0.4388	0.6200	Toxin	Rejected
**MC160L**	E3	CTSAGKGEDLATSDLTDSEPEDS	23	1.0290	0.6400	Toxin	Rejected

### Prediction of CTL epitopes

The prioritized candidate proteins were further analyzed for CTL epitopes using the NetMHCpan 4.1 EL algorithm, and 18 high-affinity CTL epitopes were identified. Each epitopes exhibited strong predicted binding to representative HLA class I molecules; their percentile ranks of binding affinities ranged from 0.01 to 0.05. Four epitopes had especially low percentile ranks, each at 0.01: HELPRPLVL from MC008L, VPVDARRAL from MC016L, APRARRGTL from MC057L, and SILRSVHEK from MC114R. The findings point to very strong predicted binding affinity and suggest that MHC class I molecules may present these epitopes efficiently. The selected CTL epitopes cover a broad set of HLA alleles: HLA-A11:01, HLA-B07:02, HLA-B40:01, HLA-B53:01, and HLA-A*68:01. This would increase the likelihood of broad immune recognition across genetically diverse populations. These epitopes were then considered for incorporation into the multi-epitope vaccine construct. The detailed prediction results are presented in Table 9.

**Table 9:** Selected CTL epitopes predicted by NetMHCpan 4.1 EL from the prioritized molluscum contagiosum virus proteins.

Protein	CTL epitope	Start–end	HLA allele	Percentile rank	Binding
**MC005L**	SQFLELAIF	44–52	HLA-B*15:01	0.02	Strong binder
**MC005L**	ASLGEVAGK	72–80	HLA-A*11:01	0.03	Strong binder
**MC005L**	MEALVQMRL	25–33	HLA-B*40:01	0.05	Strong binder
**MC008L**	HELPRPLVL	153–161	HLA-B*40:01	0.01	Strong binder
**MC008L**	CVFPVPEAR	162–170	HLA-A*68:01	0.02	Strong binder
**MC008L**	VPTSFVFRF	121–129	HLA-B*53:01	0.02	Strong binder
**MC016L**	VPVDARRAL	164–172	HLA-B*07:02	0.01	Strong binder
**MC016L**	SLYSTTNSV	22–30	HLA-A*02:03	0.01	Strong binder
**MC016L**	ASAALSTLY	2–10	HLA-A*30:02	0.01	Strong binder
**MC057L**	APRARRGTL	47–55	HLA-B*07:02	0.01	Strong binder
**MC057L**	VSDTLWLRY	63–71	HLA-A*01:01	0.01	Strong binder
**MC057L**	YVSDTLWLR	62–70	HLA-A*68:01	0.02	Strong binder
**MC114R**	SILRSVHEK	37–45	HLA-A*11:01	0.01	Strong binder
**MC114R**	SMYRTIRQY	211–219	HLA-B*15:01	0.01	Strong binder
**MC114R**	TPALNITLF	284–292	HLA-B*53:01	0.02	Strong binder
**MC160L**	SEHEVLRFL	21–29	HLA-B*40:01	0.02	Strong binder
**MC160L**	QVAAINNMV	97–105	HLA-A*68:02	0.02	Strong binder
**MC160L**	IPFSFLRNL	6–14	HLA-B*51:01	0.02	Strong binder

### Prediction of HTL epitopes

The prioritized candidate proteins were analyzed using the IEDB NetMHCIIpan 4.1 EL algorithm, and high-affinity HTL epitopes with low percentile rank values indicating strong predicted binding to HLA class II molecules were identified. The top-scoring epitopes that did not overlap from different proteins are selected to maximize immune stimulation and minimize sequence redundancy. The top-ranked non-overlapping HTL epitopes, together with their core peptides, corresponding HLA alleles, and percentile ranks, are summarized in Table 10. The HTL epitope derived from MC057L, TLWLRYDARARAVRV, received the highest score, with a percentile rank of 0.14 for HLA-DRB1*03:01. Other top-ranking non-overlapping epitopes came from MC114R, MC016L, MC160L, and MC008L, ensuring broad representation of multiple HLA-DR alleles. Only non-overlapping epitopes with a score above a defined threshold were selected. It helps in providing broad coverage of the HLA-DR super type. These, along with other details of associated antigens and epitopes, are described in the subsequent section.

**Table 10:** Top-ranked helper T lymphocyte (HTL) epitopes predicted using the IEDB NetMHCIIpan 4.1 EL algorithm from the prioritized molluscum contagiosum virus proteins.

Protein	HTL epitope^a^	Core peptide	HLA allele	Percentile rank
**MC057L**	TLWLRYDARARAVRV	LRYDARARA	HLA-DRB1*03:01	0.14
**MC057L**	DTLWLRYDARARAVR	LRYDARARA	HLA-DRB1*03:01	0.16
**MC114R**	TRLGISNTPALNITL	ISNTPALNI	HLA-DRB1*07:01	0.41
**MC114R**	KRVVIVNYASMNQVP	IVNYASMNQ	HLA-DRB1*15:01	0.41
**MC057L**	SDTLWLRYDARARAV	LRYDARARA	HLA-DRB1*03:01	0.45
**MC057L**	SRWTLRADDERERRM	LRADDERER	HLA-DRB1*03:01	0.57
**MC016L**	SQRPAFVPVDARRAL	FVPVDARRA	HLA-DRB1*07:01	0.62
**MC160L**	MDVLEVDDAEPMEAT	LEVDDAEPM	HLA-DRB1*03:01	0.67
**MC016L**	LSQRPAFVPVDARRA	FVPVDARRA	HLA-DRB1*07:01	0.99
**MC008L**	LPAIVRDLGLAGEMR	IVRDLGLAG	HLA-DRB1*03:01	1.10

a Only the highest-scoring non-overlapping HTL epitopes were selected for each candidate protein. Selection was based on low percentile rank, high prediction score, representation of multiple HLA class II alleles, and suitability for downstream vaccine design. Lower percentile rank values indicate stronger predicted binding affinity.

### Population coverage analysis

**Figure 2:**
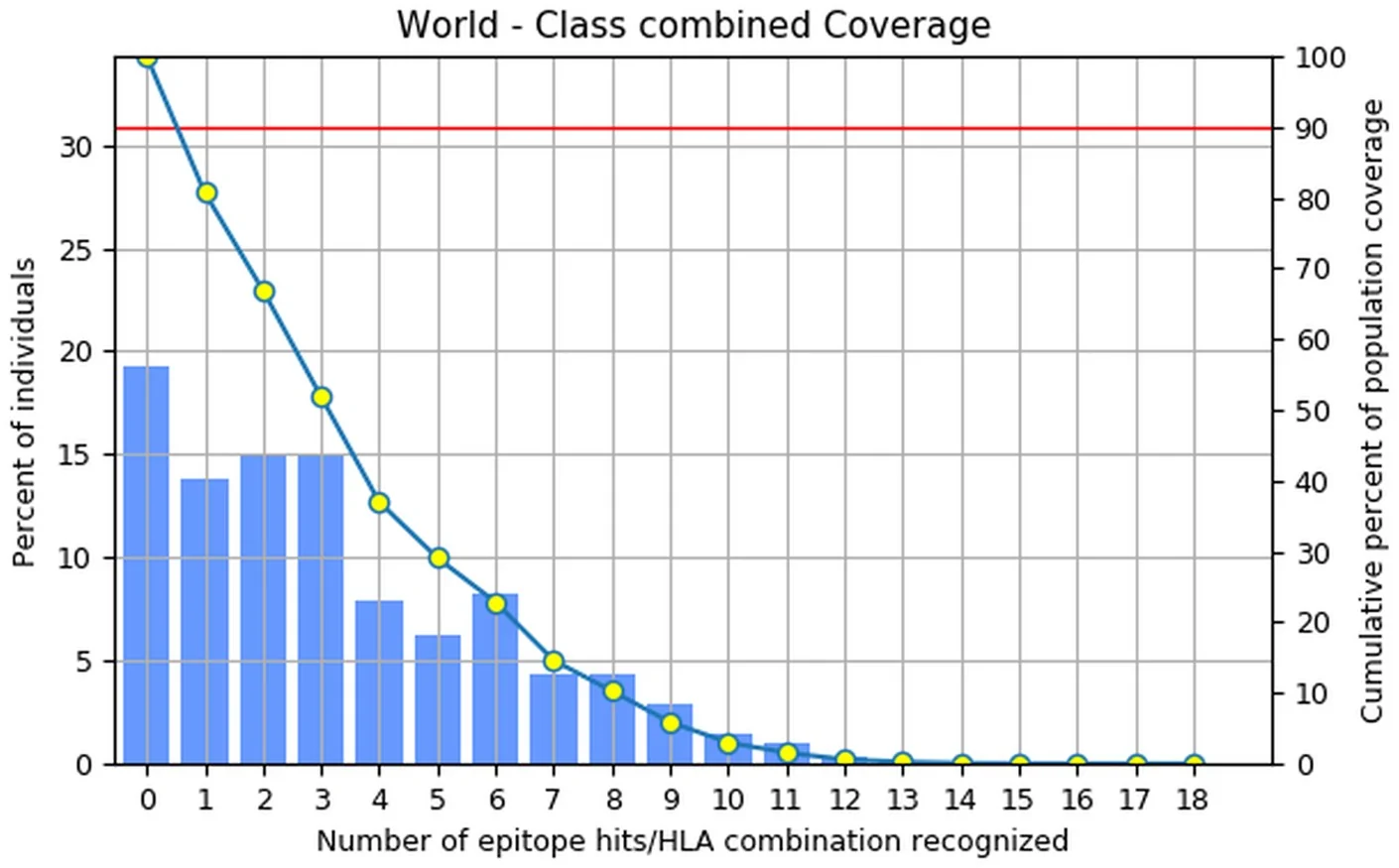
Worldwide population coverage of the selected CTL and HTL epitopes predicted using the IEDB population coverage tool

To assess the likely global applicability of the vaccine, coverage of the population was analyzed using the selected CTL and HTL epitopes and their corresponding HLA class I and class II alleles. The combined epitope set achieved an estimated worldwide population coverage of 80.69%, indicating that over 80% of the population would be capable of recognizing at least one predicted epitope–HLA combination. The average number of epitope–HLA combinations per individual that could be recognized was 3.24, with a PC90 value of 0.52, indicating broad immunological responsiveness to genetically diverse populations. These findings support the suitability of the selected epitopes for the development of a globally applicable multi-epitope vaccine. The detailed population coverage results are summarized in Table 11, whereas the worldwide distribution is illustrated in Fig. 2.

**Table 11:** Population coverage analysis of the selected CTL and HTL epitopes using the IEDB population coverage tool.

Population	Coverage (%)	Average hit	PC90
**World**	80.69	3.24	0.52

### Selection of final immunodominant epitopes

Based on the combined results of antigenicity, toxicity, MHC-binding affinity, HLA diversity, and population coverage analyses, a final set of immunodominant epitopes was selected for vaccine construction. The final vaccine design incorporated five HTL epitopes, five CTL epitopes, and three B-cell epitopes, representing proteins involved in viral immune evasion, membrane fusion, and host-cell entry. These epitopes exhibited favorable immunological characteristics, including strong predicted binding to multiple HLA alleles, high antigenicity, absence of predicted toxicity, and broad worldwide population coverage. Consequently, these peptides were considered optimal candidates for inclusion in the multi-epitope vaccine construct. The final selected epitopes and their corresponding selection criteria are presented in Table 12.

**Table 12:** Final immunodominant epitopes selected for multi-epitope vaccine construction.

Immune response	Protein	Selected epitope^a^	Selection criteria
**HTL**	MC057L	TLWLRYDARARAVRV	Strong MHC-II binding, HLA-DRB1*03:01, low percentile rank
**HTL**	MC114R	TRLGISNTPALNITL	Strong MHC-II binding, HLA-DRB1*07:01
**HTL**	MC160L	MDVLEVDDAEPMEAT	Strong MHC-II binding, high prediction score
**HTL**	MC016L	SQRPAFVPVDARRAL	Strong MHC-II binding, conserved epitope
**HTL**	MC008L	LPAIVRDLGLAGEMR	High binding affinity, antigenic
**CTL**	MC016L	VPVDARRAL	Strong binder (HLA-B*07:02)
**CTL**	MC057L	APRARRGTL	Strong binder (HLA-B*07:02)
**CTL**	MC114R	SILRSVHEK	Strong binder (HLA-A*11:01)
**CTL**	MC160L	SEHEVLRFL	Strong binder (HLA-B*40:01)
**CTL**	MC008L	HELPRPLVL	Strong binder (HLA-B*40:01)
**B-cell**	MC008L	FQEDYHVGET	Antigenic, non-toxic
**B-cell**	MC016L	RKLSLYSTTNSV	Antigenic, non-toxic
**B-cell**	MC114R	TDIANEYAVTTFSEDGYPCNKNYEITSG	Antigenic, non-toxic

a The final immunodominant epitopes were selected based on antigenicity, non-allergenicity, non-toxicity, MHC binding affinity, HLA diversity, and worldwide population coverage to maximize the immunogenic potential of the multi-epitope vaccine.

### Construction of the multi-epitope vaccine

The selected HTL, CTL, and B-cell epitopes were used to design a multi-epitope vaccine construct by linking them serially with appropriate peptide linkers. The 50S ribosomal protein L7/L12 was incorporated in the construct at the N-terminal position as an adjuvant to enhance immunogenicity. It was connected to the epitope region by an EAAAK linker. To maintain structural integrity and facilitate antigen processing, the epitopes were linked with GPGPG, AAY, and KK linkers for HTL, CTL, and B-cell epitopes, respectively. Finally, a 6 × His tag was added at the C-terminus to facilitate purification of the recombinant protein in future experimental studies. Table 13 gives a summary of the structural components of the designed vaccine construct.

**Table 13:** Components incorporated into the final multi-epitope vaccine construct.

Component	Selected element	Function
**Adjuvant**	50S ribosomal protein L7/L12, UniProt P9WHE3	Immunostimulatory adjuvant; commonly used as a TLR4-stimulating adjuvant in multi-epitope vaccine designs
**Adjuvant linker**	EAAAK	Separates the adjuvant from the epitope region
**HTL linker**	GPGPG	Separates helper T-cell epitopes
**CTL linker**	AAY	Supports proteasomal processing of CTL epitopes
**B-cell linker**	KK	Maintains flexibility between B-cell epitopes
**Purification tag**	6 × His tag	Facilitates recombinant protein purification

### Physicochemical properties of the designed vaccine construct

Physicochemical properties of the final vaccine construct were assessed using the ProtParam server to determine its biochemical suitability for recombinant expression. The designed construct was 351 amino acids long, with a predicted molecular weight of 37.48 kDa and a theoretical isoelectric point (pI) of 6.10. According to its instability index (33.29), this construct was classified as stable. The aliphatic index reading was 88.26, indicating favorable thermostability. Furthermore, the negative GRAVY value (−0.201) indicated an overall hydrophilic nature, which may enhance protein solubility. Taken together, these physicochemical properties hint that the designed vaccine would have favorable downstream production and experimental evaluation. The full physicochemical profile is given in Table 14.

**Table 14:** Physicochemical properties of the final multi-epitope vaccine construct predicted using ExPASy ProtParam.

Property	Value
**Length (amino acids)**	351
**Molecular weight (kDa)**	37.48
**Theoretical pI**	6.10
**Negatively charged residues (Asp + Glu)**	49
**Positively charged residues (Arg + Lys)**	44
**Extinction coefficient (M⁻¹ cm⁻¹)**	21 890
**Estimated half-life (mammalian cells)**	30 h
**Estimated half-life (yeast)**	>20 h
**Estimated half-life (*Escherichia coli*)**	>10 h
**Instability index**	33.29 (stable)
**Aliphatic index**	88.26
**GRAVY**	−0.201

### Secondary structure prediction

**Figure 3:**
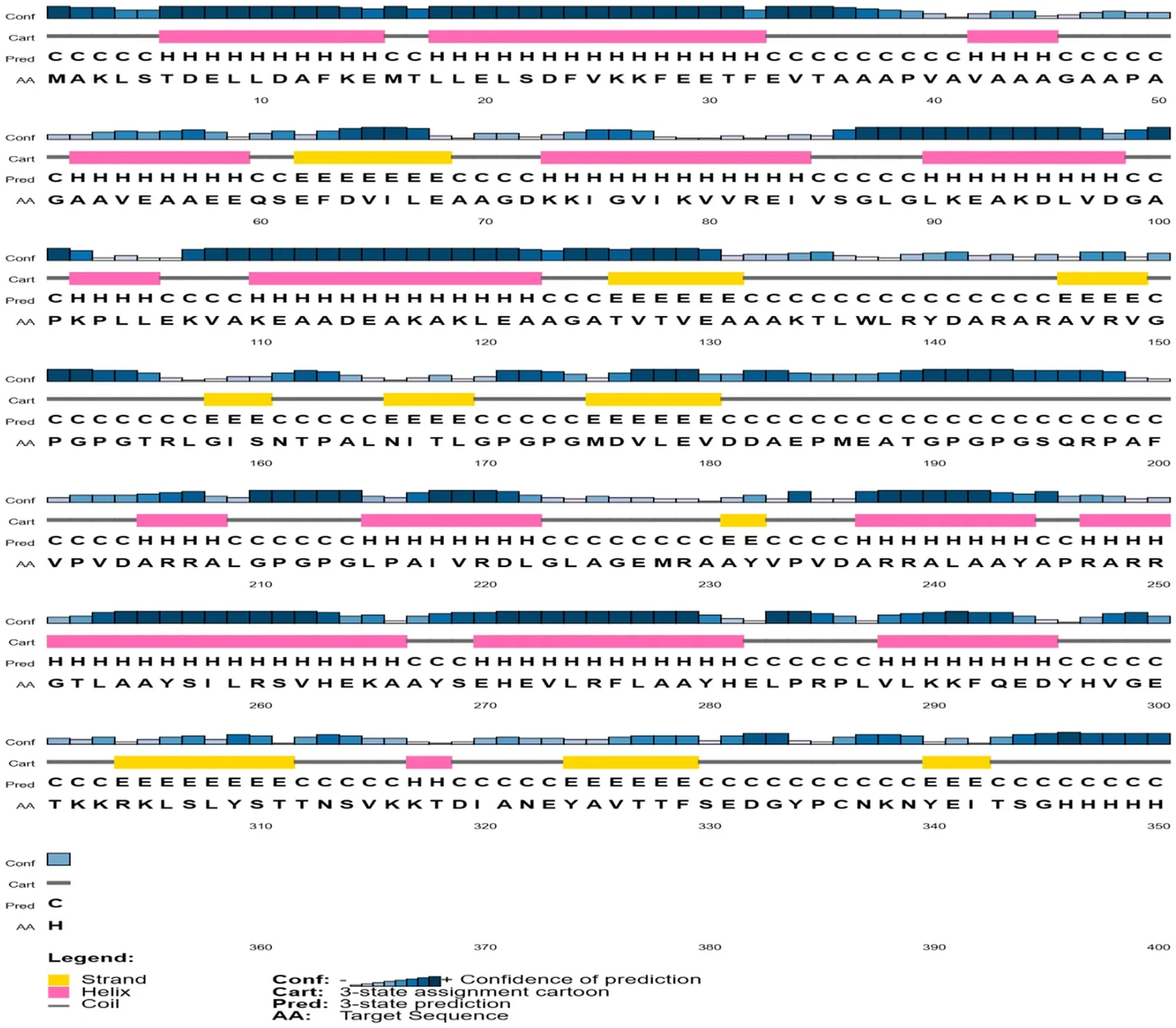
Predicted secondary structure of the final multi-epitope vaccine construct generated using the PSIPRED server

The PSIPRED server was used for the prediction of secondary structure to evaluate the structural organization of the designed multi-epitope vaccine. The model showed prediction having a balanced distribution of α-helices, β-strands, and random coils, indicating a secondary structure model organized enough to support proper folding into these regions. More helix was mainly found in the adjuvant domain, while β-strands and coils, giving flexibility and possible epitope accessibility, were found across the epitope-containing regions. The predicted secondary structure is shown in Fig. 3.

### 3D structure prediction

**Figure 4:**
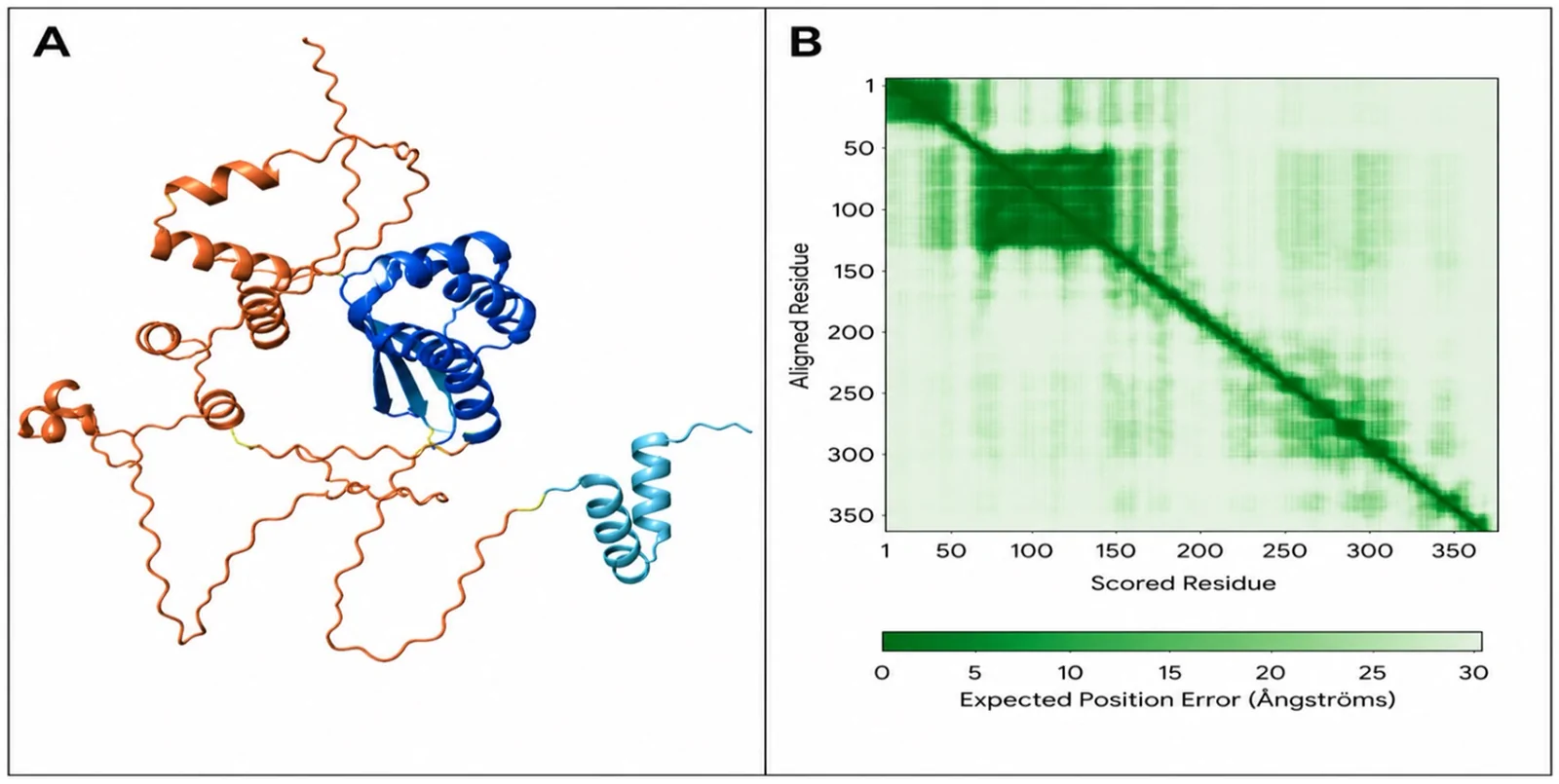
Structural prediction of the final multi-epitope vaccine construct using AlphaFold 3. (**A**) Predicted three-dimensional structure of the vaccine model. (**B**) Predicted aligned error (PAE) map showing the expected positional error between residue pairs. Dark green regions indicate lower predicted alignment error and higher confidence in the relative positioning of residues, whereas lighter green regions represent regions with greater structural flexibility

The final vaccine construct was used to predict its 3D structure using AlphaFold 3 to assess whether it had an overall structural organization and confidence. The model predicted showed a compact folded conformation with well-defined structural domains, which points to the successful structural assembly of the multi-epitope construct. The analysis of the Predicted Aligned Error (PAE) map showed low error values for most residue pairs, indicating high confidence in the predicted relative positioning of amino acid residues. Slightly higher PAE values were found in the flexible linker regions, as expected due to their inherent structural flexibility. The predicted 3D model and its corresponding PAE map are presented in Fig. 4.

### Structural validation of the vaccine construct

The predicted tertiary structure of the multi-epitope vaccine construct was further validated using PROCHECK, ERRAT, and Verify3D servers to evaluate its stereochemical quality and structural reliability. Ramachandran plot analysis generated by PROCHECK demonstrated that 82.3% of residues were located in the most favored regions, 16.7% in additionally allowed regions, and 1.0% in generously allowed regions, while no residues were detected in disallowed regions, indicating acceptable stereochemical quality of the predicted model.

**Figure 5:**
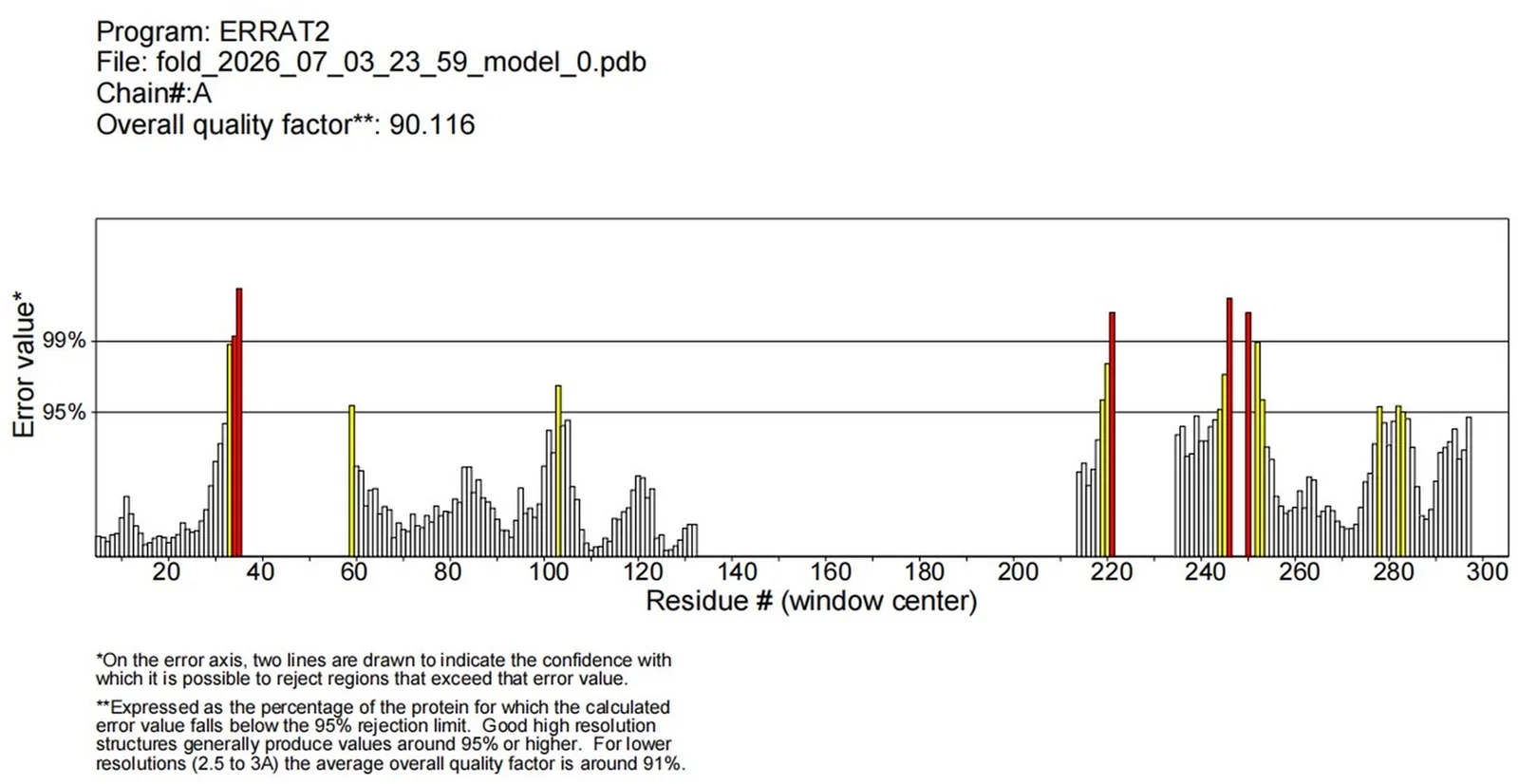
Structural quality assessment of the vaccine construct using the ERRAT server showing an overall quality factor of 90.12, indicating high reliability of the predicted three-dimensional structure. ^a^On the error axis, two lines are drawn to indicate the confidence with which it is possible to reject regions that exceed that error value. ^b^Expressed as the percentage of the protein for which the calculated error value falls below the 95% rejection limit. Good high-resolution structures generally produce values around 95% or higher. For lower resolutions (2.5 to 3A), the average overall quality factor is around 91%.

**Figure 6:**
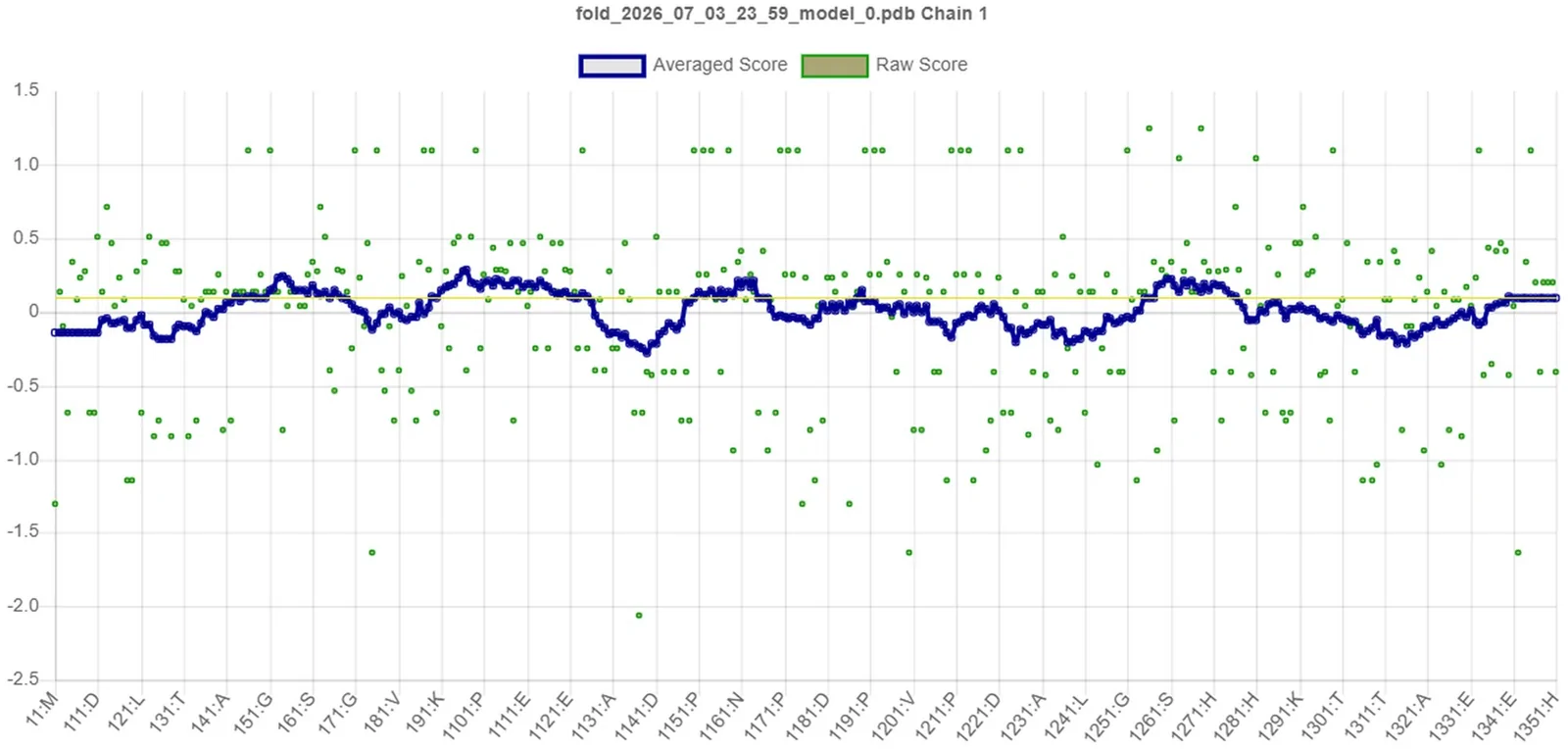
Verify3D profile analysis of the multi-epitope vaccine construct showing sequence–structure compatibility across amino acid residues

Furthermore, ERRAT analysis yielded an overall quality factor of 90.12, supporting the high reliability and structural integrity of the vaccine construct. Verify3D analysis showed partial compatibility between the amino acid sequence and the predicted 3D structure, with lower compatibility scores mainly observed within linker and flexible regions, which are commonly present in multi-epitope vaccine constructs, as shown in Fig. 5. Overall, these validation analyses support the structural quality and suitability of the predicted vaccine model for subsequent molecular docking studies, as shown in Fig. 6.

### Molecular docking analysis

To investigate the potential interaction between the designed vaccine and innate immune receptors, molecular docking was performed against the human TLR4 receptor using the HDOCK server. Among the generated docking models, Model 1 achieved the best docking performance with a docking score of −190.46 and a confidence score of 0.6919, indicating a favorable binding interaction. The receptor template exhibited 100% sequence identity and complete structural coverage, while the ligand showed 34.8% template coverage with 50.8% sequence identity. Interaction analysis identified five hydrogen bond/salt bridge interactions and five electrostatic interactions, supporting the formation of a stable vaccine–TLR4 complex. These docking results suggest that the designed vaccine may effectively interact with TLR4 and potentially stimulate innate immune signaling. The docking parameters are summarized in Table 15.

**Table 15:** Molecular docking results and interaction analysis of the designed multi-epitope vaccine with the human TLR4 receptor.

Parameter	Value
**Docking server**	HDOCK
**Receptor**	Human Toll-like receptor 4 (TLR4, PDB ID: 4G8A, Chain A)
**Ligand**	Designed multi-epitope vaccine construct
**Selected docking model**	Model 1
**Docking score**	**−190.46**
**Confidence score**	**0.6919**
**Receptor template coverage**	**100%**
**Receptor sequence identity**	**100%**
**Ligand template**	**2ZJQ (Chain 5)**
**Ligand template coverage**	**34.8%**
**Ligand template sequence identity**	**50.8%**
**Number of hydrogen bond/salt bridge interactions**	**5**
**Number of electrostatic interactions**	**5**
**Hydrogen bond distance range**	**2.76–3.56 Å**
**Electrostatic interaction distance range**	**4.05–5.44 Å**
**Major interacting receptor residues**	Lys31, Lys79, Arg82, Lys104, Lys127, Arg32, Arg35, Glu59
**Interaction type**	Hydrogen bonds, salt bridges, electrostatic interactions
**Selected for further analysis**	Model 1

Note: Bold values indicate the key docking and template-quality parameters used to characterize the selected docking model.

### Protein–protein interaction analysis

**Figure 7:**
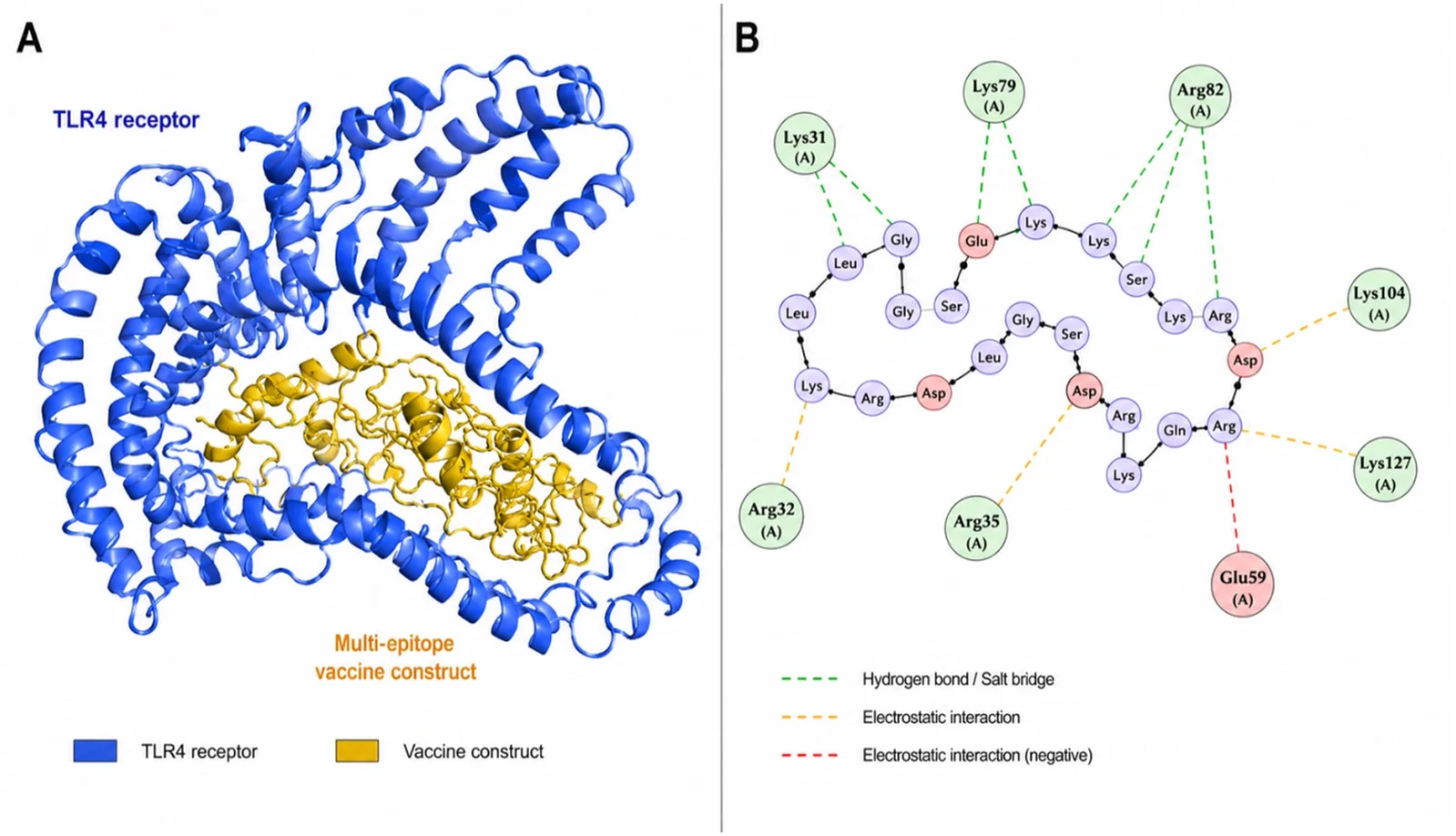
Molecular docking and interaction analysis of the designed multi-epitope vaccine with the human toll-like receptor 4 (TLR4)

Detailed visualization of the vaccine–TLR4 docking complex revealed several non-covalent interactions that contributed to complex stabilization. The selected docking model contained five hydrogen bond/salt bridge interactions with Lys31, Lys79, and Arg82, exhibiting interaction distances ranging from 2.76 to 3.56 Å, consistent with stable hydrogen bonding. Additionally, five electrostatic interactions involving Lys104, Lys127, Arg32, Arg35, and Glu59 were identified, with interaction distances between 4.05 and 5.44 Å. These complementary intermolecular interactions indicate a stable binding interface between the vaccine construct and TLR4, supporting the predicted ability of the vaccine to activate receptor-mediated innate immune responses. The detailed interaction profile is presented in Table 16, whereas the 3D docking complex and interaction network are illustrated in Fig. 7.

**Table 16:** Detailed protein–protein interactions between the designed multi-epitope vaccine and the human TLR4 receptor.

No.	Interaction type	Receptor residue	Distance (Å)
**1**	Hydrogen bond (salt bridge)	Lys31	2.97
**2**	Hydrogen bond (salt bridge)	Lys79	3.46
**3**	Hydrogen bond (salt bridge)	Lys79	3.19
**4**	Hydrogen bond (salt bridge)	Arg82	2.76
**5**	Hydrogen bond (salt bridge)	Arg82	3.56
**6**	Electrostatic	Lys104	4.87
**7**	Electrostatic	Lys127	4.05
**8**	Electrostatic	Arg32	5.44
**9**	Electrostatic	Arg35	5.35
**10**	Electrostatic	Glu59	5.43

### Molecular dynamics simulation of the vaccine–TLR4 complex using iMODS

**Figure 8:**
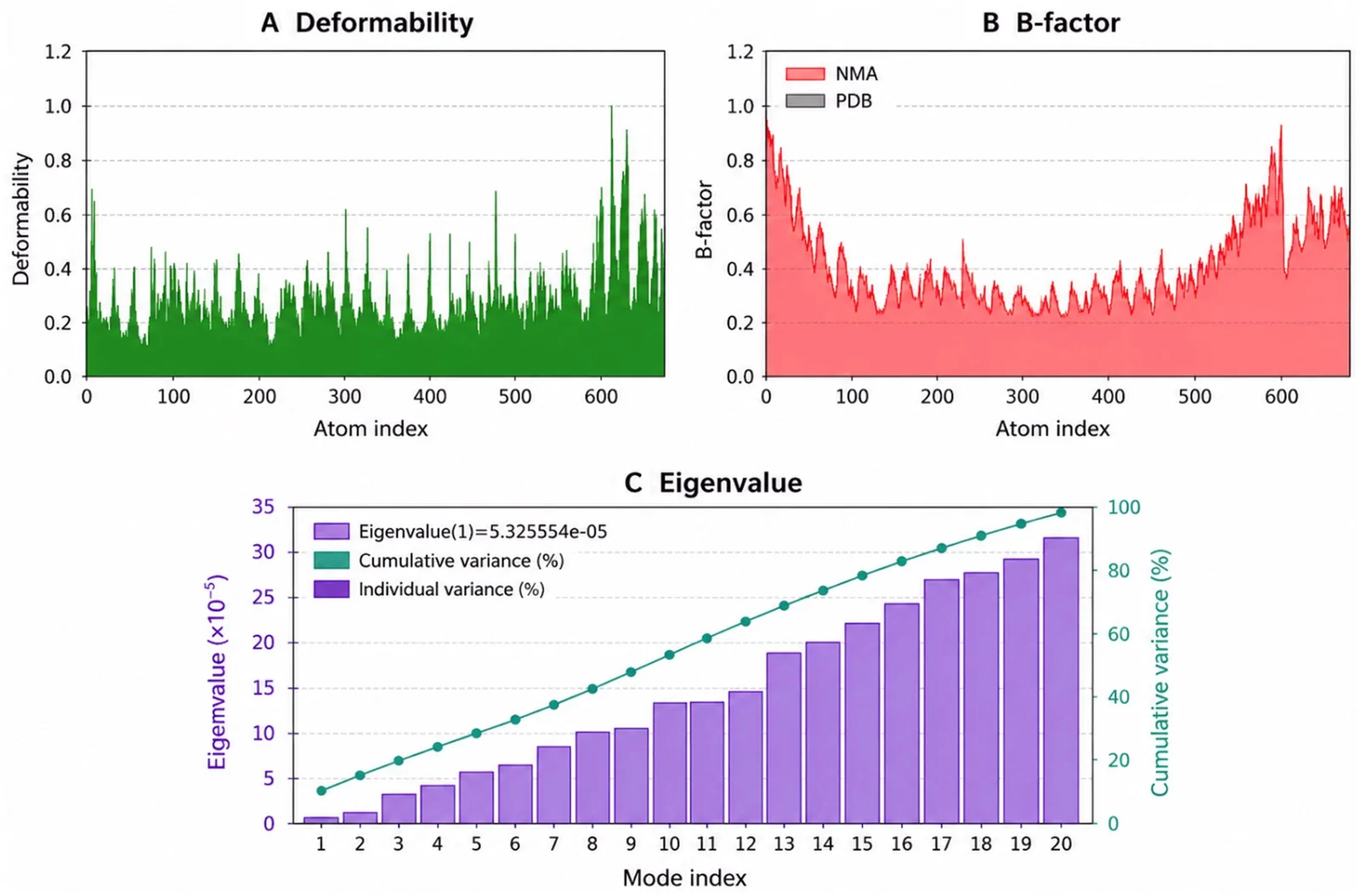
Flexibility and mobility analysis of the vaccine–TLR4 complex generated using iMODS showing (**A**) deformability profile, (**B**) B-factor distribution, and (**C**) eigenvalue analysis of the docked complex

**Figure 9:**
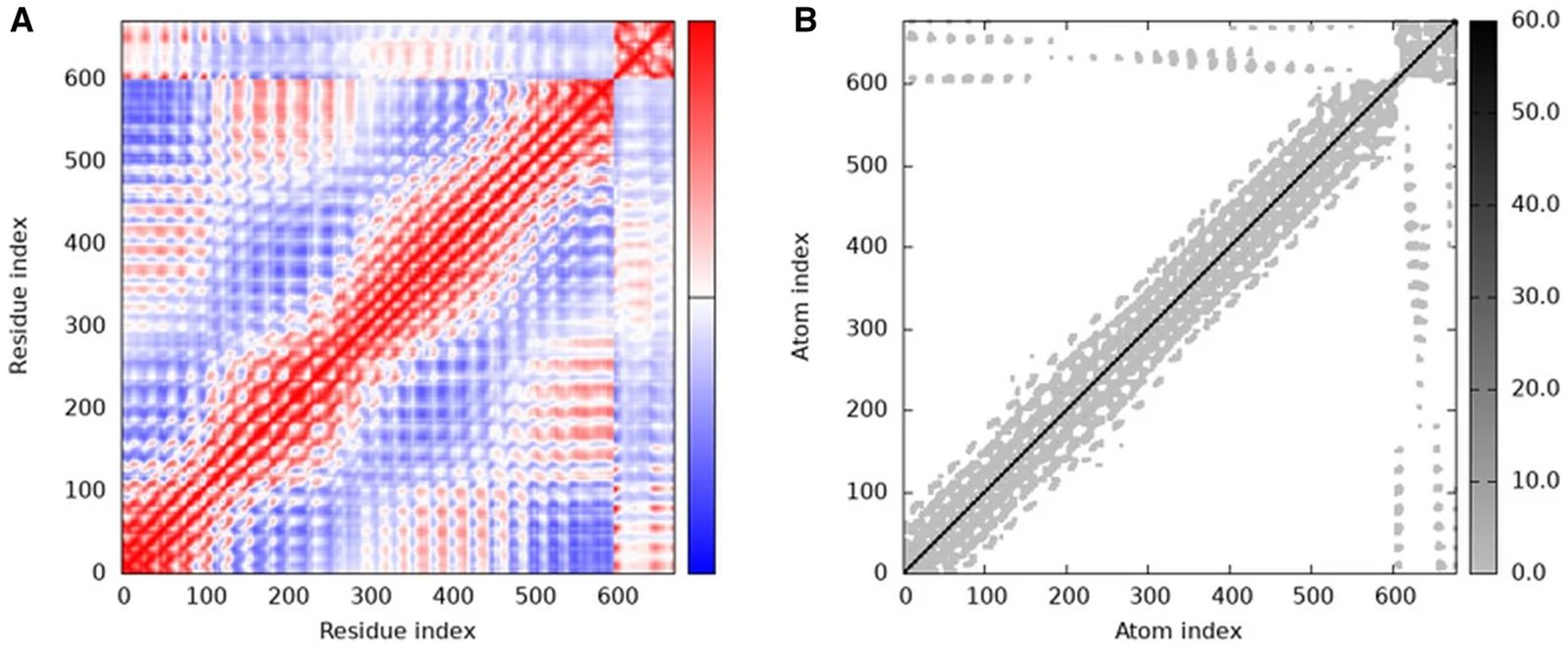
Dynamic interaction analysis of the vaccine–TLR4 complex generated using iMODS showing (**A**) covariance matrix and (**B**) elastic network model of the docked complex

To further evaluate the stability and flexibility of the docked vaccine–TLR4 complex, normal mode analysis (NMA) was performed using the iMODS server. The deformability analysis demonstrated limited flexibility across most regions of the complex, with higher mobility observed only in a few loop regions, indicating overall structural stability as shown in Fig. 8. The B-factor profile showed a mobility pattern consistent with the deformability results. Moreover, eigenvalue analysis revealed that a considerable amount of energy is required to deform the complex, supporting the rigidity and stability of the interaction between the vaccine construct and TLR4 receptor. Overall, these findings suggest that the docked complex possesses favorable dynamic stability and may maintain stable interactions under physiological conditions, as shown in Fig. 9. iMODS was chosen to assess the collective motions and mechanical flexibility of the large protein complex while keeping the computational demands manageable.

## Discussion

### Candidate protein selection

The success of reverse vaccinology depends on the selection of target proteins that are biologically relevant and immunologically important. In this study, protein prioritization was based on predicted antigenicity, accessibility to the host immune system, and established roles in viral pathogenesis and immune evasion. A number of the selected proteins such as MC005L, MC159L, MC160L, and MC114R have been reported to play a role in either host antiviral response modulation or viral morphogenesis, therefore making them attractive vaccine targets [8, 15]. MC005L has been shown to block NF-κB activation by binding to the host NEMO signaling complex, contributing to the virus’s suppression of inflammatory responses during infection [29]. In a similar fashion, MC159L expresses a viral FLIP protein that inhibits apoptosis and regulates inflammatory signaling pathways; MC160L further suppresses the activation of innate immunity by inhibiting cGAS-STING-mediated interferon responses [35, 37]. In contrast, MC114R is considered a structural protein because it plays a role in virion morphogenesis and viral particle assembly; thus, it is a suitable target for antibody-mediated immune responses [38].

### Physicochemical characterization and topology analysis

The physicochemical properties of the vaccine targets are very important in their suitability for vaccine development. In the present study, it was observed that the selected proteins had favorable molecular weights, stability indices, and hydrophilicity profiles, which indicate their potential suitability for recombinant expression and downstream vaccine construction. This was in line with the findings of Gasteiger *et al.* [14] where ProtParam analysis was used to determine such parameters. Topology prediction across the membrane also indicated that most proteins selected had either no transmembrane helices or very few regions spanning the membrane, thus favoring access to epitopes and immune recognition. In previous studies, it has been demonstrated that proteins with large transmembrane regions pose difficulties for recombinant production and epitope presentation in subunit vaccine design [18].

### Epitope prediction and immunogenicity assessment

The induction of both humoral and cellular components of the immune response, humoral and cellular, is a prerequisite for the development of successful antiviral vaccines. Thus, B-cell, cytotoxic T lymphocyte (CTL), and helper T lymphocyte (HTL) epitopes were identified using validated immunoinformatics tools to maximize immune coverage and efficacy of the vaccine. The selected B-cell epitopes were predicted to contribute to antibody recognition, whereas the CTL and HTL epitopes were predicted to stimulate cellular immune responses through binding to MHC class I and class II molecules, respectively. Collectively, these complementary immune components are expected to enhance the breadth of the predicted vaccine-induced immune response [39]. Only epitopes showing high antigenicity while also being non-allergenic and non-toxic were considered for the vaccine formulation to increase the expected safety profile of the final vaccine candidate [18, 19, 22].

### Population coverage analysis

Major limitation of peptide-based vaccines is the extensive polymorphism of human leukocyte antigen (HLA) molecules among different populations. Therefore, an analysis of population coverage was carried out to estimate the proportion of individuals who would be able to present the selected epitopes. The predicted global population coverage obtained in this study suggests that the selected epitope set may allow for broad immunological applicability in multiple geographic regions and ethnic groups. Such broad HLA representation is considered a crucial factor in maximizing vaccine effectiveness at the population level [24].

### Multi-epitope vaccine construction

The final vaccine construct was made by rational assembly of selected B-cell, CTL, and HTL epitopes using appropriate peptide linkers to preserve epitope integrity and help in processing and presentation of the antigen. Multi-epitope vaccine strategies have come up as better alternatives to conventional vaccines because of their safety and specificity in addition to their ability to raise balanced humoral and cellular immune responses [40]. The physicochemical analysis of the final construct showed a positive molecular weight, stability index, antigenicity score, and solubility characteristics, which further supported the potential suitability of this construct for recombinant production and future experimental validation.

### Structural modeling and validation

Reliable structural modeling is also needed to assess the conformational stability of vaccine constructs and predict interactions with receptors. In this study, we used AlphaFold 3 to build a high-confidence 3D model of the vaccine candidate [28].

The model’s stereochemical quality was checked through PROCHECK, ERRAT, and Verify3D. Ramachandran statistics showed that most residues were in favored regions. The ERRAT quality factor was above the generally accepted threshold for high-quality models. Verify3D results showed only some deviations in local residue environments, a finding often seen in large chimeric vaccine constructs made using computational methods and do not necessarily compromise their overall structural reliability [41, 42].

### Molecular docking analysis

The docking of the compound with innate immune receptors was considered important for the initiation of a strong antiviral immune response. Toll-like receptor 4 (TLR4) was chosen as the docking receptor because it has a well-known function in the recognition of pathogen-associated molecular patterns and subsequent activation of inflammatory signaling pathways[43]. The docking analysis showed favorable binding affinity and multiple intermolecular interactions between the vaccine construct and TLR4. This suggests that the vaccine may have the ability to promote innate immune activation, which subsequently enhances adaptive immune responses [44].

### Normal mode analysis

The dynamic stability of the vaccine-TLR4 complex was further investigated with normal mode analysis using the iMODS server. The low eigenvalue seen for the complex shows that very little energy would be needed to prompt conformational motions, suggesting favorable flexibility and structural adaptability during receptor interaction. Deformability profiles also showed that there was flexibility at specific sites, mainly in the loop regions. Covariance and elastic network analyses indicated that there were residues with cooperative motions that maintained the overall structure of the complex. Taken together, these results point to a stable and biologically meaningful interaction between the vaccine candidate and the TLR4 receptor [45].

### Experimental support for multi-epitope vaccine strategies

Although the present MCV vaccine construct was assessed only through computational analyses, recent experimental work offers proof of concept that immunoinformatics-guided, multi-epitope designs can produce measurable immune responses once formulated as peptides, recombinant proteins, or DNA vaccines. In a SARS-CoV-2 study, Kibria et al. created a conserved multi-epitope subunit vaccine using epitopes from the envelope, membrane, and spike proteins. Mice that received the vaccine developed significantly higher levels of antigen-specific IgG, while sera collected from the immunized animals displayed virus-neutralizing activity in a Vero-cell cytopathic-effect inhibition assay [46]. The results showed that epitopes chosen according to computational binding, conservation, and population-coverage criteria could remain immunogenic after experimental formulation. Still, the authors stressed that further safety studies were needed, along with direct testing of protection against viral replication in target tissues. Guo *et al.* [47] provided additional experimental support by using immunoinformatics to identify conserved CTL epitopes in the HPV16 E5, E6, and E7 proteins. Vaccination with the resulting multi-epitope peptide and recombinant protein constructs elicited antigen-specific interferon-γ responses in mice. In the TC-1 tumor model, prophylactic immunization completely prevented tumor development, while therapeutic vaccination significantly slowed tumor growth and extended animal survival. This model addressed HPV-associated antitumor immunity rather than protection from acute viral infection, but it still showed that computationally prioritized CTL epitopes can be processed *in vivo* and produce biologically functional cellular immune responses.

Atanasova *et al.* [48] later tested a multi-epitope SARS-CoV-2 vaccine prototype containing predicted MHC class I- and class II-binding peptides in human ACE2-transgenic mice. The vaccine changed lymphocyte profiles and led T cells to produce significant amounts of IFN-γ and IL-4, but the humoral response remained relatively weak. That result matters because it demonstrates that accurate computational prediction and receptor-binding analysis do not necessarily produce equally strong humoral and cellular responses. The final immune profile is also shaped by the formulation, adjuvant, epitope combination, dose, and delivery platform. More recently, Leal *et al.* [49] developed multi-epitope DNA vaccine constructs containing conserved epitopes from the Zika virus envelope and NS1 proteins, then evaluated them in BALB/c mice. Both constructs increased CD4⁺ and CD8⁺ T-lymphocyte populations and induced IgG responses associated with a Th1 immune profile. When fused to the ssPGIP signal peptide, the construct produced a broader response, with greater antibody production and stimulation of cytokines linked to Th1, Th2, and Th17 pathways. The findings underscore how intracellular antigen delivery and signal-peptide engineering can influence the processing and presentation of a computationally designed multi-epitope antigen to the immune system. Taken together, these experimental studies show that combining predicted B-cell, CTL, and HTL epitopes in a single vaccine formulation is biologically feasible. They also show where immunoinformatics is useful: it can help prioritize antigens, but it cannot stand in for experimental testing. The evidence cited above concerns SARS-CoV-2, HPV16, and Zika virus, not MCV, so it supports the broader multi-epitope vaccine strategy rather than the efficacy of the present MCV construct. For that reason, the current docking, structural-validation, and normal-mode analysis results should be treated as hypothesis-generating evidence. Establishing whether the proposed MCV vaccine can produce protective immunity *in vivo* will require recombinant expression, confirmation of epitope processing and HLA presentation, assessment of cytokine and antibody responses, cytotoxicity and allergenicity testing, and, ultimately, challenge studies.

### Study limitations and future perspectives

Although the present study provides a comprehensive computational framework for MCV vaccine design, several limitations should be acknowledged. First, all findings were generated using *in silico* prediction tools and therefore require experimental validation using *in vitro* and *in vivo* models. Second, normal mode analysis provides an approximation of molecular flexibility and cannot fully substitute for long-timescale molecular dynamics simulations.

Future studies should focus on recombinant expression of the vaccine construct, immunogenicity testing in animal models, and evaluation of protective efficacy against MCV infection. Such investigations will be essential for translating the proposed vaccine candidate into practical clinical applications.

## Conclusion

This study applied a comprehensive reverse vaccinology and immunoinformatics pipeline to systematically identify vaccine candidates against MCV. From the complete MCV proteome, a few selected proteins prioritized based on their participation in immune evasion, viral entry, and virion assembly processes were subjected to extensive computational analyses. The final multi-epitope vaccine construct contained epitopes of HTL, CTL, and B cells selected on the basis of their favorable antigenicity, non-allergenicity, non-toxicity, and broad HLA population coverage. Results from structural modeling and validation tests indicate that the design of our construct was stable and reliable. Additionally, our results from molecular docking and normal mode analyses suggest that our construct should have a strong and stable interaction with the human TLR4 receptor. In conclusion, the proposed vaccine construct should be considered a promising candidate for preventing MCV infection and will provide an invaluable computational base for future vaccine development efforts. But, further experimental validations, including *in vitro* immunological assays and *in vivo* animal studies, are needed to confirm the predicted immunogenicity, safety, and protective efficacy of the vaccine before human use.

## Supplementary Material

bpag045_Supplementary_Data

## Data Availability

All data generated or analyzed during this study are included in this published article and its Supplementary Material. Any additional information related to the study is available from the corresponding author upon reasonable request.
